# Dataset on substituents effect on biological activities of linear RGD-containing peptides as potential anti-angiotensin converting enzyme

**DOI:** 10.1016/j.dib.2023.109478

**Published:** 2023-08-06

**Authors:** Abel Kolawole Oyebamiji, Sunday Adewale Akintelu, Emmanuel Temitope Akintayo, Cecillia Olufunke Akintayo, Halleluyah O. Aworinde, Oluwatobi D. Adekunle

**Affiliations:** a Computational Chemistry Research Laboratory, Industrial Chemistry Programme, Bowen University, Iwo, Osun State, Nigeria; b Department of Pure and Applied Chemistry, Ladoke Akintola University of Technology, P.M.B. 4000, Ogbomoso, Oyo State, Nigeria; c Department of Chemistry, Ekiti State University, Ado-Ekiti, Nigeria; d Department of Chemistry, Federal University, Oye-Ekiti, Nigeria; e College of Computing and Communication Studies, Bowen University, Iwo, Nigeria

**Keywords:** Peptides, RGD, Angiotensin converting enzyme, Diabetes, Disease, DFT, *In silico*

## Abstract

The angiotensin converting enzyme inhibiting activity of linear rgd-containing peptides was investigated using *in silico* approach. The synthesized compound (parent compound) using experimental approach as well as its derivatives was subjected to computational examination using appropriate software. The investigated compounds were optimized using Spartan 14 while the docking study was executed via Pymol, AutoDock Tool, AutoDock Vina and discovery studio. The descriptors obtained (2D and 3D) were screened and the descriptor with highest capacity (squared correlation coefficient) was correlated to the calculated binding affinity. More so, the docking analysis was performed on the investigated linear rgd-containing peptides and angiotensin converting enzyme (PDB ID: 3nxq) via docking software and the resulted scoring and the types of the interaction observed were presented. Furthermore, (S)-dimethyl 2-(2-((S)-2-((R)-1-((S)-2-((S)-2-((S)-3-(4-chlorophenyl)-2-(1,3-dioxoisoindolin-2-yl)propanamido)-4-(methylthio)butanamido)-4-methylpentanoyl)pyrrolidine-2-carboxamido)-5-(3-((2,2,4,5,7-pentamethyl-2,3-dihydrobenzofuran-6-yl)sulfonyl)guanidino)pentanamido)acetamido)succinate (AB5) (compound with lowest binding affinity) and metformin were subjected to ADMET analysis and the resulted outcome were reported appropriately.

Specification Table

**Table:** 

Subject	Combinatorial Study
Specific subject area	Drug synthesis and prediction
Type of data	Figure Table ADMET Arithmetic model
How data were acquired	Solid phase peptide synthesis, Spartan 14, PADEL, Pymol 1.7.4.4, AutoDock Tool 1.5.6; AutoVina, Discovery studio
Data format	Raw Data
Description of data collection	The peptide was synthesized from solid phase peptide synthesis using 2-Chlorotrityl chloride resin. The coupling of the Fmoc-protected amino acids were achieved with Oxyma, NMP and DIC as coupling agent, 20% of piperidine in DMF was used for the deprotection of Fmoc and the peptide was cleaved from resin via 2% trifluoracetic acid in DCM. The crude peptide was esterified with thionyl chloride in methanol to obtain the final product. Six (6) derivatives were added to the synthesized compound and were optimized via Spartan 14 tool. Features obtained which expressed the activities of the selected compounds were used as independent variables which helped in quantitative structure activity relationship model development using Genetic function Algorithm (GFA) embedded in material studio software. The synthesized compound and the other derivatives were docked into the active site of angiotensin converting enzyme (PDB ID: 3nxq) via docking method. The obtained results were reported and interpreted.
Data source location	Computational Chemistry Research Laboratory, Industrial Chemistry Programme, Bowen University, Iwo, Osun State, Nigeria
Data accessibility	The experimental and predicted data can be accessed in the data article (https://data.mendeley.com/datasets/npdyw6dh25/2); doi:10.17632/npdyw6dh25.2

## 1 Value of the Data

• The chemical shift obtained from the HNMR will enable the scientists to understand the position and number of the Hydrogen atoms present in the peptide.
• It helps researchers in ascertaining and elucidating the right structure of the peptide.
• The generated data from the 3D structure of the linear RGD-containing peptides will expose scientists to their anti-angiotensin converting enzyme activities.
• The calculated 2-dimensional and 3-dimensional descriptors from the optimized compounds will give scientists better understanding about appropriate features that describes anti-angiotensin converting enzyme activities of linear RGD-containing peptides.
• The calculated binding affinity from the docked complexes will reveal to researchers compounds with superior tendency to inhibit angiotensin converting enzyme (PDB ID: 3nxq).
• The level of absorption and its ability to act as drug via ADMET analysis will help scientists to know the probable action of individual molecule (drug-like molecules) in human being.

## 2 Objective

To investigate the anti-angiotensin converting enzyme activity of the linear rgd-containing peptides via quantum chemical and molecular modelling investigations using *in silico* approach.

## 3 Data Description

Table 1 showed the combination of three dimensional structures (optimized using Spartan 14 software [1,2]), two-dimensional structure and the IUPAC names of the investigated compounds.

**Table 1:** 3D and 2D configurations of investigated linear rgd-containing peptides.

	3D Structure		IUPAC Name
AB1			(S)-dimethyl 2-(2-((S)-2-((R)-1-((S)-2-((S)-2-((S)-2-(1,3-dioxoisoindolin-2-yl)propanamido)-4-(methylthio)butanamido)-4-methylpentanoyl)pyrrolidine-2-carboxamido)-5-(3-((2,2,4,5,7-pentamethyl-2,3-dihydrobenzofuran-6-yl)sulfonyl)guanidino)pentanamido) acetamido)succinate
AB2			(S)-dimethyl 2-(2-((S)-2-((R)-1-((S)-2-((S)-2-((S)-2-(1,3-dioxoisoindolin-2-yl)-3-(p-tolyl)propanamido)-4-(methylthio)butanamido)-4-methylpentanoyl)pyrrolidine-2-carboxamido)-5-(3-((2,2,4,5,7-pentamethyl-2,3-dihydrobenzofuran-6-yl)sulfonyl)guanidino)pentanamido) acetamido)succinate
AB3			(S)-dimethyl 2-(2-((S)-2-((R)-1-((S)-2-((S)-2-((S)-2-(1,3-dioxoisoindolin-2-yl)-3-(4-methoxyphenyl)propanamido)-4-(methylthio)butanamido)-4-methylpentanoyl)pyrrolidine-2-carboxamido)-5-(3-((2,2,4,5,7-pentamethyl-2,3-dihydrobenzofuran-6-yl)sulfonyl)guanidino)pentanamido) acetamido)succinate
AB4			(S)-dimethyl 2-(2-((S)-2-((R)-1-((S)-2-((S)-2-((S)-2-(1,3-dioxoisoindolin-2-yl)-3-(4-(methoxycarbonyl)phenyl)propanamido)-4-(methylthio)butanamido)-4-methylpentanoyl)pyrrolidine-2-carbo xamido)-5-(3-((2,2,4,5,7-pentamethyl-2,3-dihydrobenzofuran-6-yl)sulfonyl)guanidino)pentanamido)acetamido)succinate
AB5			(S)-dimethyl 2-(2-((S)-2-((R)-1-((S)-2-((S)-2-((S)-3-(4-chlorophenyl)-2-(1,3-dioxoisoindolin-2-yl)propanamido)-4-(methylthio)butanamido)-4-methylpentanoyl)pyrrolidine-2-carboxamido)-5-(3-((2,2,4,5,7-pentamethyl-2,3-dihydrobenzofuran-6-yl)sulfonyl)guanidino)pentanamido) acetamido)succinate
AB6			(S)-dimethyl 2-(2-((S)-2-((R)-1-((S)-2-((S)-2-((S)-2-(1,3-dioxoisoindolin-2-yl)-3-(4-fluorophenyl)propanamido)-4-(methylthio)butanamido)-4-methylpentanoyl)pyrrolidine-2-carboxamido)-5-(3-((2,2,4,5,7-pentamethyl-2,3-dihydrobenzofuran-6-yl)sulfonyl)guanidino)pentanamido) acetamido)succinate
AB7			(S)-dimethyl 2-(2-((S)-2-((R)-1-((S)-2-((S)-2-((S)-3-(4-bromophenyl)-2-(1,3-dioxoisoindolin-2-yl)propanamido)-4-(methylthio)butanamido)-4-methylpentanoyl)pyrrolidine-2-carboxamido)-5-(3-((2,2,4,5,7-pentamethyl-2,3-dihydrobenzofuran-6-yl)sulfonyl)guanidino)pentanamido) acetamido)succinate

Two and three dimensional descriptors obtained from the investigated linear rgd-containing peptides were revealed in Tables 2 and 3. Several descriptors obtained from both 2 and 3-dimensional structures of the investigated compounds were observed and vetted. The three dimensional descriptors were dipole moment, highest occupied molecular orbital energy, molecular weight, area, lowest unoccupied molecular orbital energy, band-gap, polar surface area, log P, hydrogen bond donor and hydrogen bond acceptor whereas two dimensional features were ATS1p, ATS2p, ATS4p, ATS5p, AATS1e, AATS3e, AATS1s, AATS2s, AATS3s and AATS4s

**Table 2:** Features (Descriptors) for 3-dimensional configuration of investigated linear rgd-containing peptides.

	E_HOMO_	E_LUMO_	BG	DM	MW	AREA	VOL	PSA	OVA	LOG P	POL	HBD	HBA
AB1	-8.81	-1.84	6.97	5.21	1169.389	1156.79	1134.16	249.981	2.2	0.76	131.73	5	23
AB2	-6.56	-0.99	5.57	8.62	1259.514	1206.7	1226.22	240.844	2.18	2.93	139.53	5	23
AB3	-6.37	-1.35	5.02	7.23	1275.513	1216.88	1233.85	248.646	2.19	2.31	140.28	5	24
AB4	-7.55	-1.78	5.77	12.45	1303.523	1220.78	1252.7	262.271	2.17	2.26	141.63	5	24
AB5	-6.73	-1.25	5.48	7.89	1279.932	1229.64	1223.16	257.026	2.22	3	139.31	5	23
AB6	-6.99	-1.47	5.52	11.67	1263.477	1193.66	1210.4	250.756	2.17	2.6	138.26	5	23
AB7	-6.99	-1.39	5.6	13.1	1324.383	1220.44	1226.21	244.498	2.2	3.27	139.52	5	23

**Table 3:** Features (Descriptors) for 2-dimensional configuration of investigated linear rgd-containing peptides.

ATS1p	ATS2p	ATS4p	ATS5p	AATS1e	AATS3e	AATS1s	AATS2s	AATS3s	AATS4s
282.0515	426.7111	566.8629	578.9491	7.862377	7.893143	3.054798	3.301921	3.318397	3.690106
311.044	471.1961	622.3625	638.5317	7.82213	7.842739	3.0247	3.234548	3.225897	3.537497
310.9338	469.3496	624.8926	645.4387	7.848863	7.869496	3.061492	3.271023	3.257313	3.574573
315.062	475.5706	631.0327	651.8273	7.859406	7.872925	3.108241	3.348448	3.321558	3.662996
308.5551	468.2251	614.5454	633.3751	7.846056	7.871906	3.036233	3.262812	3.234141	3.582407
305.8447	462.8043	609.6705	630.6646	7.854438	7.88538	3.100712	3.348398	3.330117	3.6321
310.008	471.1309	617.1585	634.828	7.841969	7.865337	3.033511	3.2592	3.23009	3.58031

**Fig. 1:**
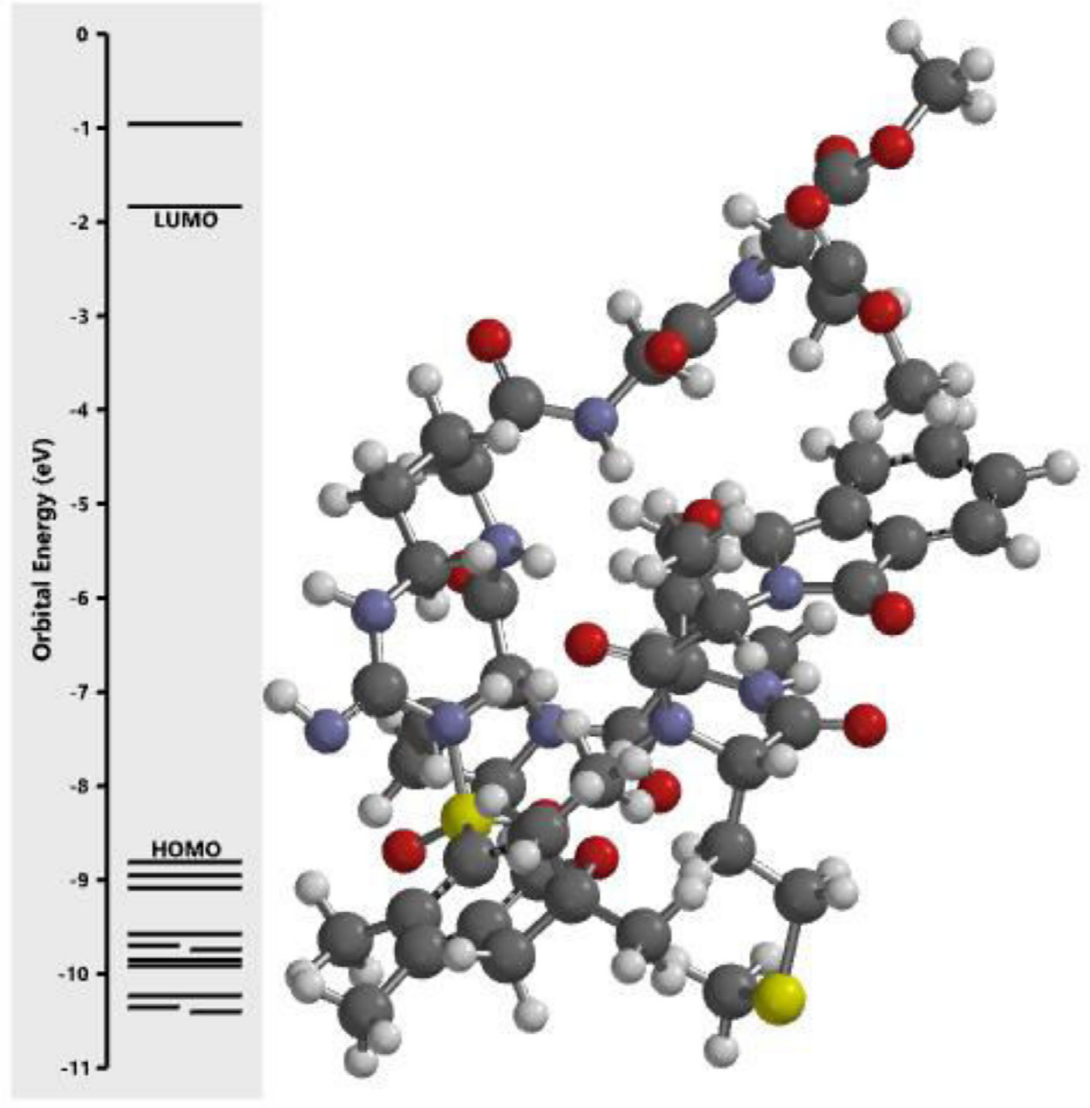
Calculated orbital energy for AB 1.

**Fig. 2:**
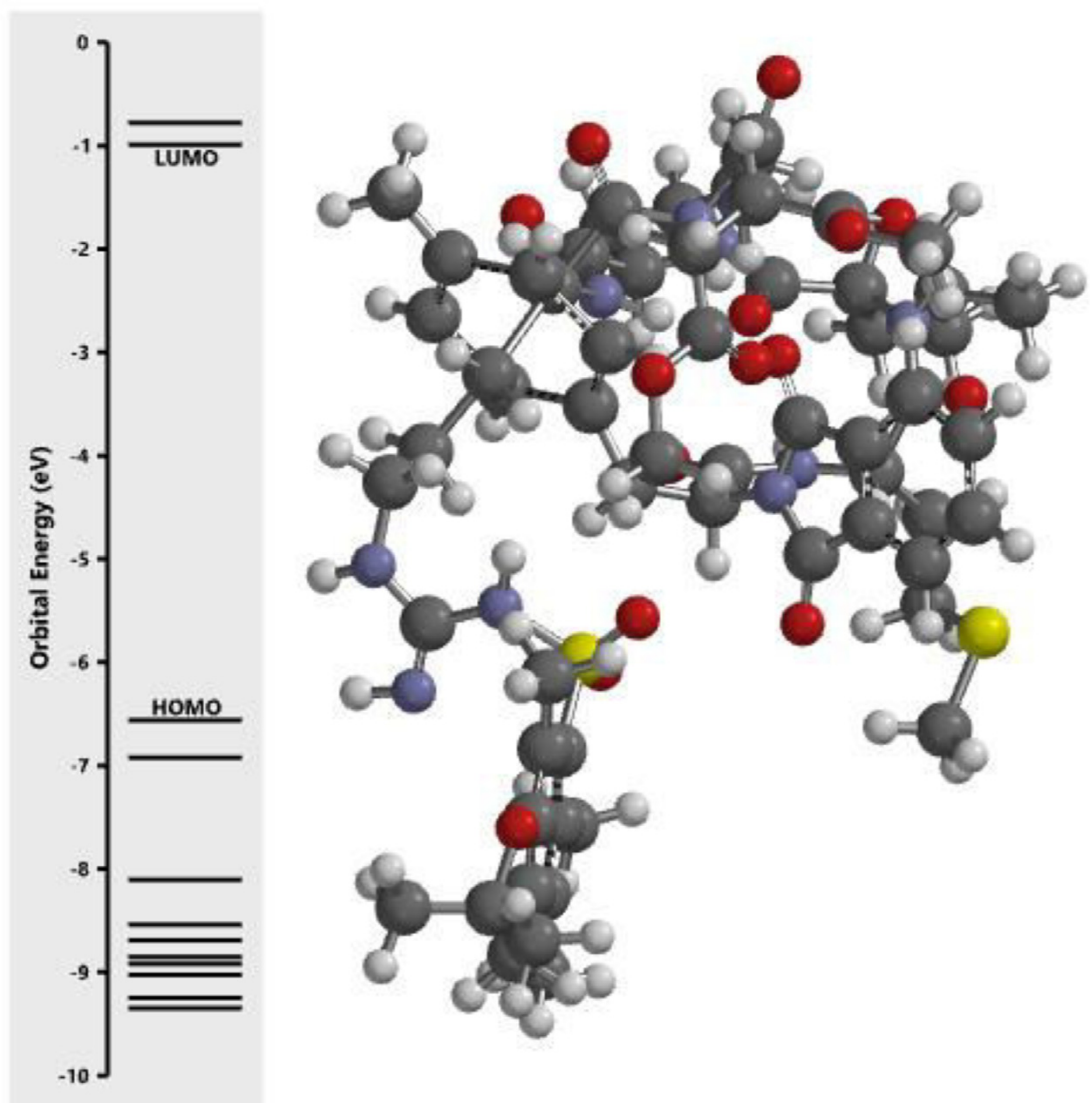
Calculated orbital energy for AB 2.

**Fig. 3:**
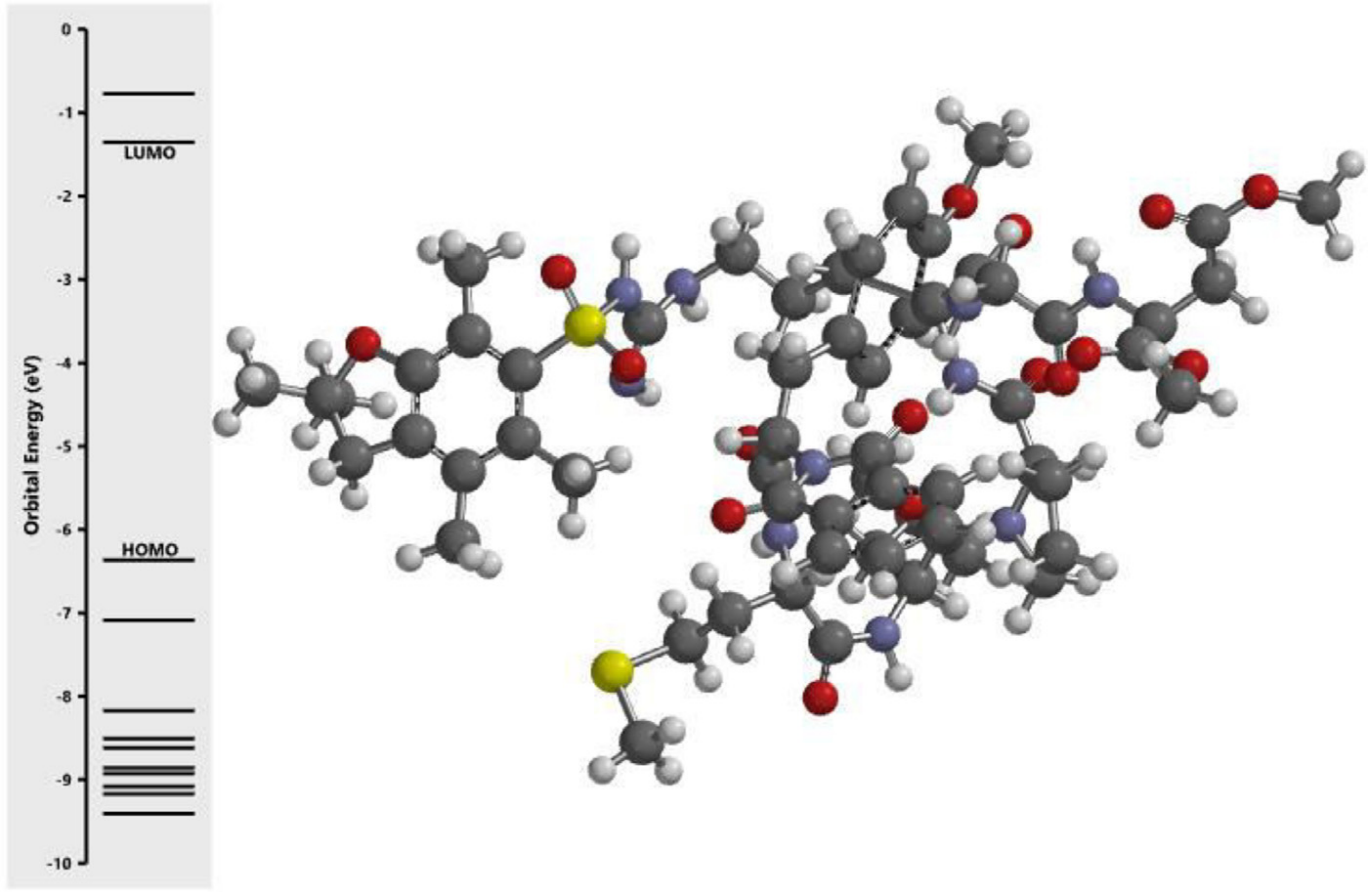
Calculated orbital energy for AB 3.

**Fig. 4:**
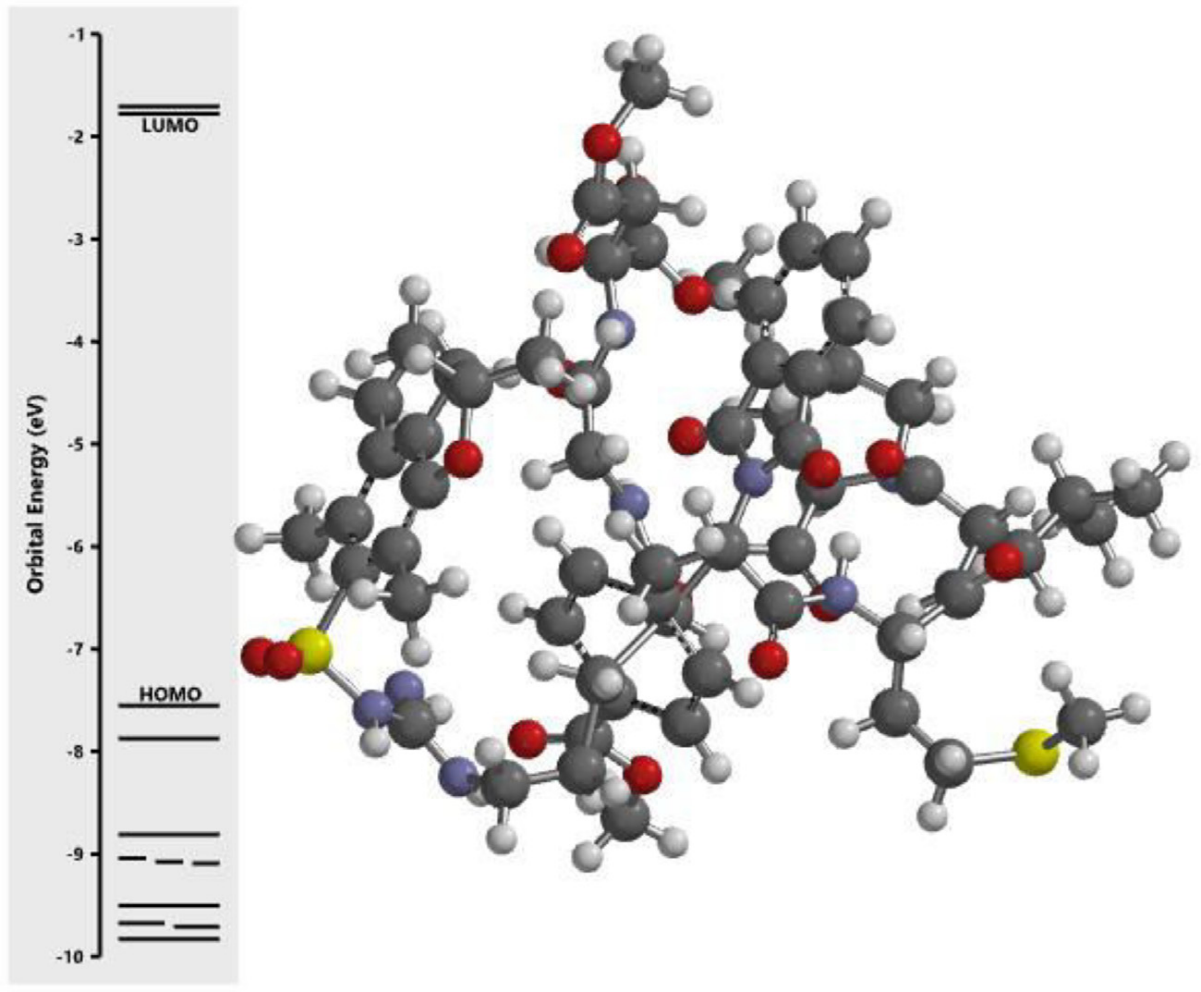
Calculated orbital energy for AB 4.

**Fig. 5:**
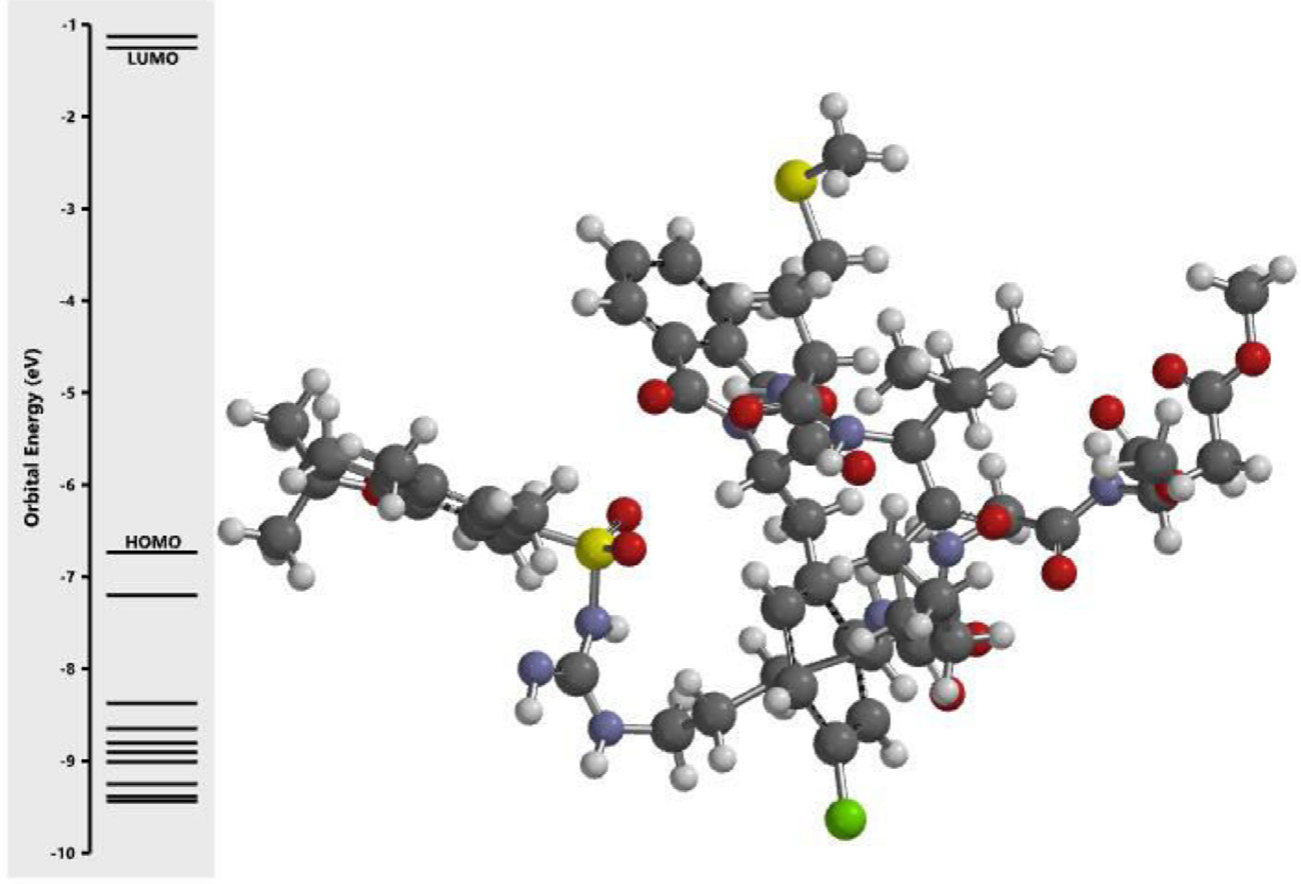
Calculated orbital energy for AB 5.

**Fig. 6:**
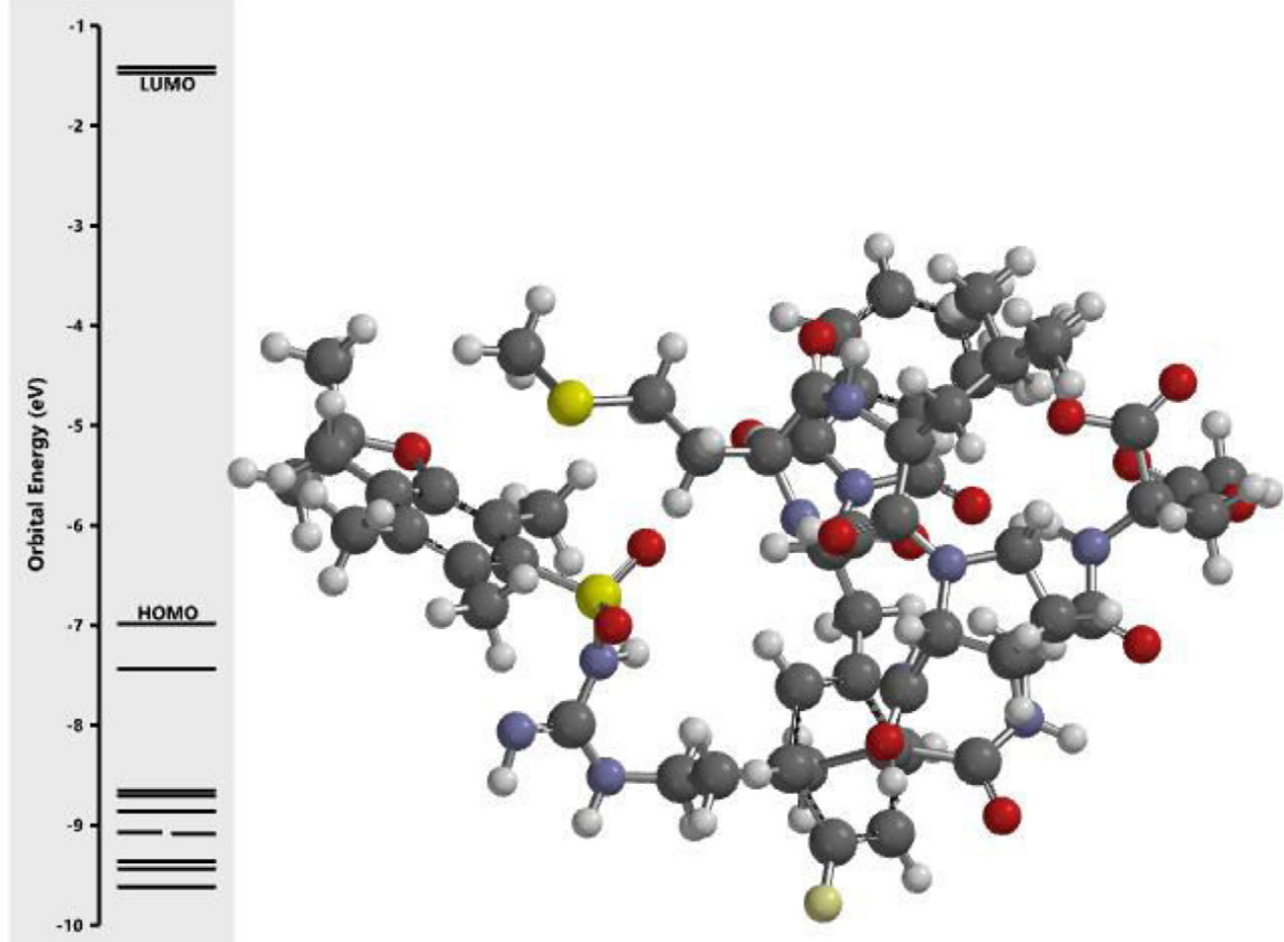
Calculated orbital energy for AB 6.

**Fig. 7:**
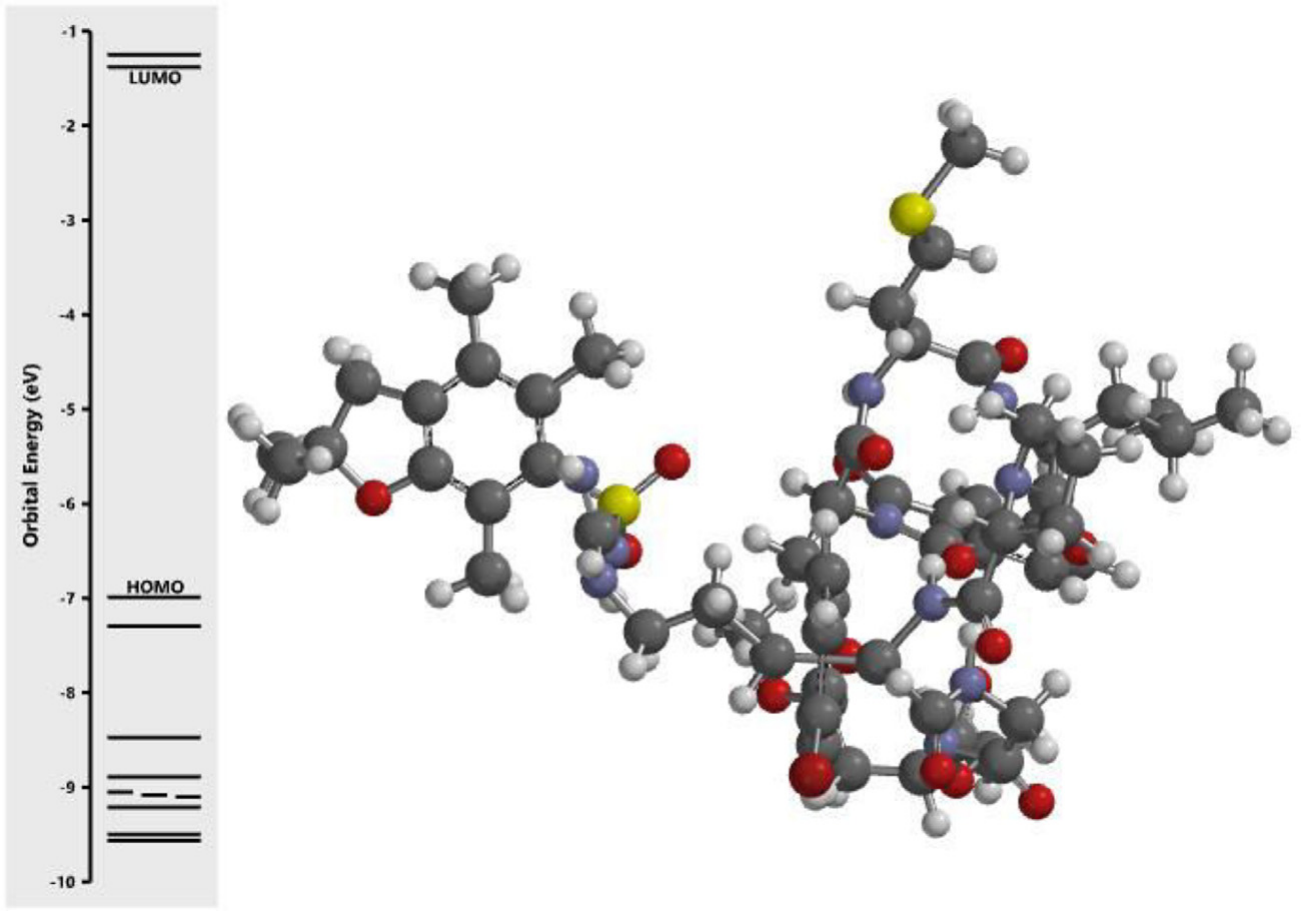
Calculated orbital energy for AB 7.

The calculated energy in each of the investigated orbital in the three dimensional structure of the examined compounds was displayed in Fig. 1, Fig. 2, Fig. 3, Fig. 4, Fig. 5, Fig. 6, Fig. 7. The scale of the E_HOMO_ and E_LUMO_ were displayed on the profile with the difference between LUMO and HOMO energies (band gap) obviously presented in Fig. 1, Fig. 2, Fig. 3, Fig. 4, Fig. 5, Fig. 6, Fig. 7. Also, the region rich in HOMO and LUMO energies in the investigated compounds were shown in Table 4. As shown in Table 2, other descriptors obtained were band gap (BG), dipole moment (DM), molecular weight (MW), Area, volume (VOL), polar surface area (PSA), ovality (Ova), lipophilicity (Log P), polarizability (Pol), hydrogen bond donor (HBD), hydrogen bond acceptor (HBA).

**Table 4:** HOMO-LUMO Overlay for the investigated phytochemicals.

	HOMO	LUMO
AB1		
AB2		
AB3		
AB4		
AB5		
AB6		
AB7		

**Fig. 8:**
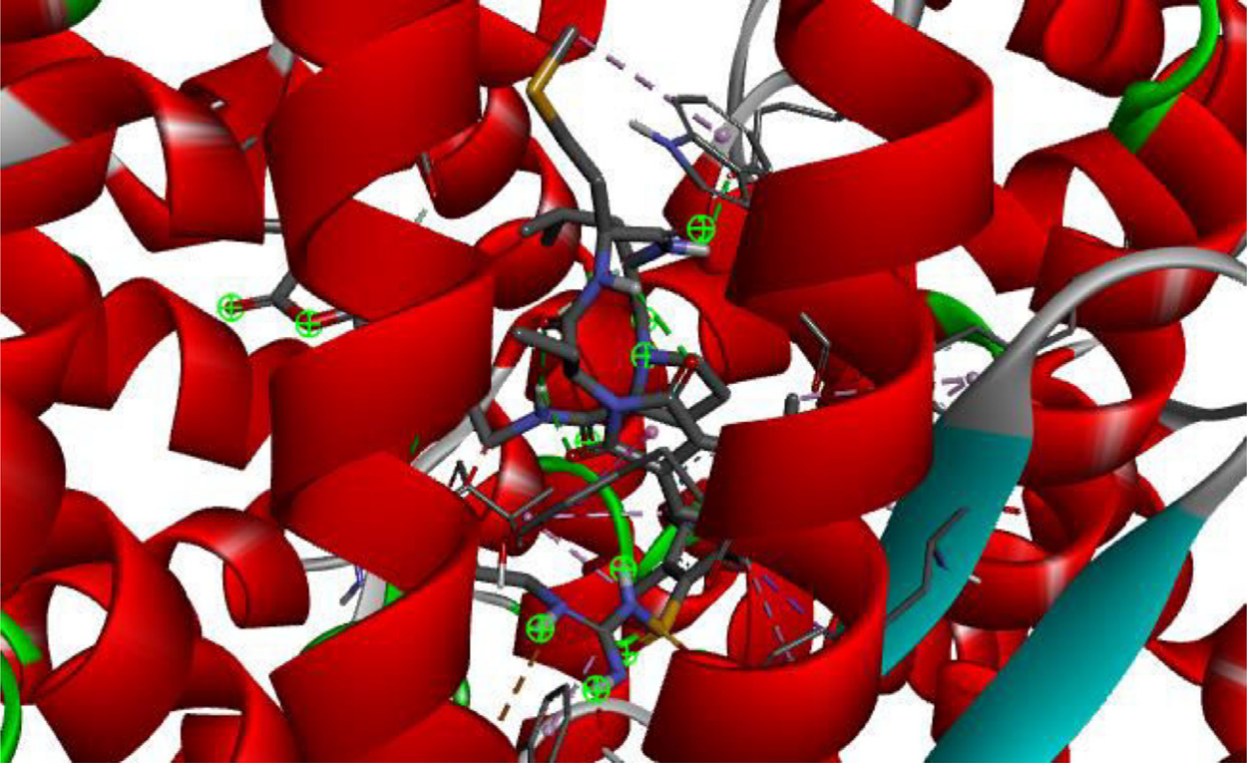
3D picture of AB5 in the binding spot of angiotensin converting enzyme (PDB ID: 3nxq).

The scoring calculated from docked complexes, the observed amino acid binding to the reacting atom in the compound as well as type of interactions observed between the interacting atoms in the investigated ligand-receptor complex were displayed in Table 5. The observed binding affinity between compound AB1-AB7 and angiotensin converting enzyme (PDB ID: 3nxq) [3] were -9.1kcal/mol for AB1, -10.1 kcal/mol for AB2, -9.3 kcal/mol for AB3, -8.8 kcal/mol for AB4, -10.4 kcal/mol for AB5, -9.1 kcal/mol for AB6, and -10.2 kcal/mol for AB7. The amino acid present in the interaction were Trp59, Ile88, Tyr360, Tyr62, Asn66, Asp358, Glu384, Ala356, Arg522, Ser355, Pro407, Met223, Ser517, Val518, Arg124, Glu123, Ile204, Ala207 for AB1; Trp59, Asn66, Ile88, Tyr62, Asp359, Arg522, His410, Gly404, Glu403, Ala63, Trp357, Arg124, Glu123 for AB2; Phe570, Met223, Trp59, Arg522, Glu403, Pro407, Trp357, Tyr360, Ala63, Asn66, Arg124 for AB3; Arg124, Arg522, Glu403, Glu123, Trp220, Phe391, Tyr62, Ala63, Asn66 for AB4; Trp59, Tyr62, Glu403, Ser517, Glu 123, Ser219 for AB5 (Fig. 8); Tyr62, Asn66, Arg124, Arg522, Glu403, Trp357, His387, Glu403 for AB6; His388, His365, His 491, Tyr 501, His361, Ala 332, Thr496, His 331, Ala334, Val329, Ser333 for AB7. The type of interactions involved in the interaction were conventional hydrogen bond, Carbon hydrogen bond, unfavourable donor-donor, Pi-Anion, Pi-Sulfur, Pi-Pi T-shaped, Alkyl, Pi-Alkyl for AB1; conventional hydrogen bond, Carbon hydrogen bond, Pi-Anion, Pi-Pi T-shaped, Alkyl, Pi-Alkyl for AB2; conventional hydrogen bond, carbon hydrogen bond, pi-cation, pi-anion, pi-pi T-shaped, alkyl, pi-alkyl for AB3; conventional hydrogen bond, carbon hydrogen bond, pi-donor hydrogen bond, pi-pi T-shaped, pi-alkyl for AB4; conventional hydrogen bond, carbon hydrogen bond, pi-pi T-shaped, pi-alkyl for AB5 (Fig. 8); conventional hydrogen bond, pi-pi T-shaped, pi-alkyl for AB6; conventional hydrogen bond, carbon hydrogen bond, pi-cation, pi-pi stacked, alkyl, pi-alkyl for AB7 (Supplementary file 1-7) and Supp 8.

**Table 5:** The observed binding affinity and amino acid residues.

	Binding Affinity (kcal/mol)	Amino Acid Residues	Non-bonding interaction
AB1	-9.1	Trp59, Ile88, Tyr360, Tyr62, Asn66, Asp358, Glu384, Ala356, Arg522, Ser355, Pro407, Met223, Ser517, Val518, Arg124, Glu123, Ile204, Ala207	Conventional hydrogen bond, Carbon hydrogen bond, unfavourable donor-donor, Pi-Anion, Pi-Sulfur, Pi-Pi T-shaped, Alkyl, Pi-Alkyl
AB2	-10.1	Trp59, Asn66, Ile88, Tyr62, Asp359, Arg522, His410, Gly404, Glu403, Ala63, Trp357, Arg124, Glu123	Conventional hydrogen bond, Carbon hydrogen bond, Pi-Anion, Pi-Pi T-shaped, Alkyl, Pi-Alkyl
AB3	-9.3	Phe570, Met223, Trp59, Arg522, Glu403, Pro407, Trp357, Tyr360, Ala63, Asn66, Arg124	Conventional hydrogen bond, Carbon hydrogen bond, Pi-Cation, Pi-Anion, Pi-Pi T-shaped, Alkyl, Pi-Alkyl
AB4	-8.8	Arg124, Arg522, Glu403, Glu123, Trp220, Phe391, Tyr62, Ala63, Asn66	Conventional hydrogen bond, Carbon hydrogen bond, Pi-Donor hydrogen bond, Pi-Pi T-shaped, Pi-Alkyl
AB5	-10.4	Trp59, Tyr62, Glu403, Ser517, Glu 123, Ser219	Conventional hydrogen bond, Carbon hydrogen bond, Pi-Pi T-shaped, Pi-Alkyl
AB6	-9.1	Tyr62, Asn66, Arg124, Arg522, Glu403, Trp357, His387, Glu403	Conventional hydrogen bond, Pi-Pi T-shaped, Pi-Alkyl
AB7	-10.2	His388, His365, His 491, Tyr 501, His361, Ala 332, Thr496, His 331, Ala334, Val329, Ser333	Conventional hydrogen bond, Carbon Hydrogen bond, Pi-Cation, Pi_pi Stacked, Alkyl, Pi-Alkyl
Metformin	-5.6	-	-

**Fig. 9:**
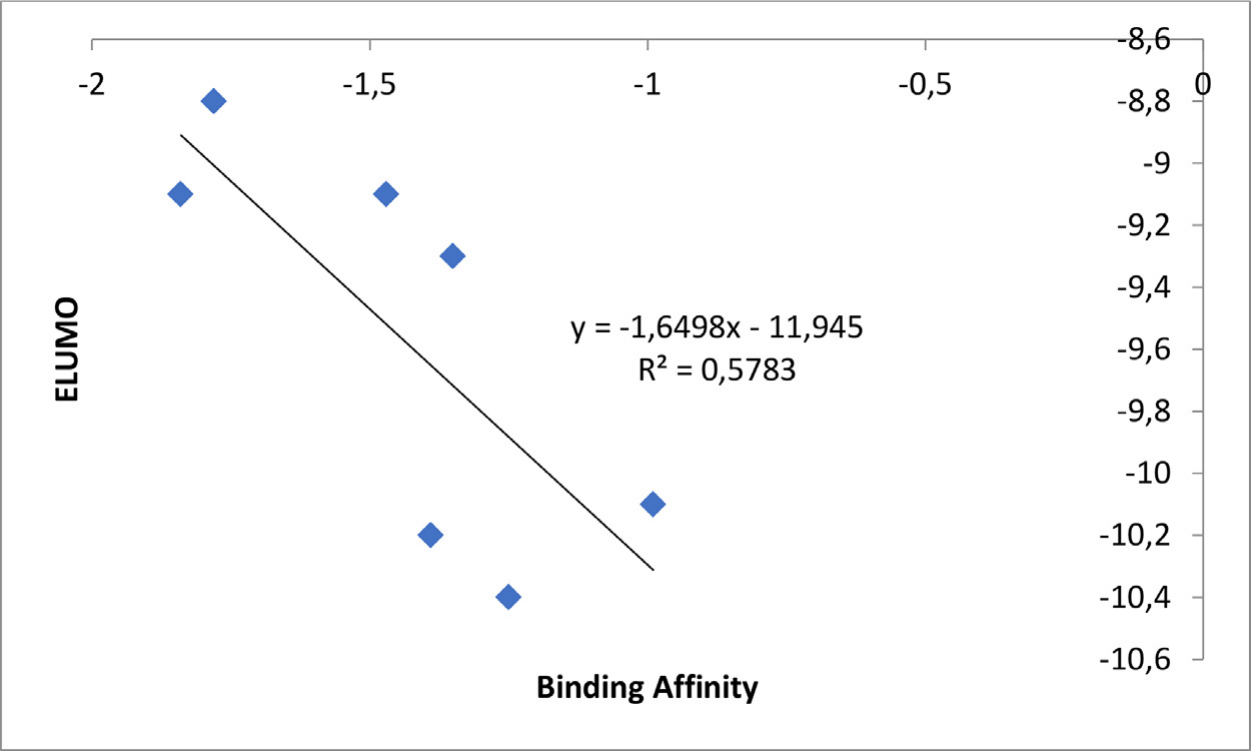
Graph of E_LUMO_ against calculated binding affinity.

Fig. 9 revealed the graph of the observed descriptor (E_LUMO_) and binding affinity. The E_LUMO_ was shown on the vertical while the binding affinity was shown on the horizontal side of the graph. The calculated squared correlation (R^2^) for the graph of E_LUMO_ and binding affinity was revealed to be 0.5783.

Tables 6 and 7 showed the absorption, distribution, metabolism, excretion and toxicity for AB5 and metformin (Reference compound) using ADMETlab [4]. The investigated factors were

**Table 6:** Observed ADMET features for AB5.

Physicochemical Property	

Molecular Weight (MW)	1278.490
Volume	1236.298
Density	1.034
nHA	25
nHD	8
nRot	37
nRing	6
MaxRing	9
nHet	28
fChar	0
nRig	44
Flexibility	0.841
Stereo Centers	6
TPSA	349.570
logS	-4.066
logP	4.704
logD	2.372

Medicinal Chemistry	

QED	0.018
SAscore	5.786
Fsp^3^	0.517
MCE-18	169.121
NPscore	-0.372
Lipinski Rule	Rejected
Pfizer Rule	Accepted
GSK Rule	Rejected
Golden Triangle	Rejected
PAINS	0 alert(s)
ALARM NMR Rule	3 alert(s)
BMS Rule	0 alert(s)
Chelator Rule	0

Absorption	

Caco-2 Permeability	-5.785
MDCK Permeability	3.1e-05
Pgp-inhibitor	+++
Pgp-substrate	+++
HIA	-
F_20%_	+++
F_30%_	+++

Distribution	

PPB	94.028%
VD	0.210
BBB Penetration	—
Fu	5.519%

Metabolism	

CYP1A2 inhibitor	—
CYP1A2 substrate	—
CYP2C19 inhibitor	—
CYP2C19 substrate	—
CYP2C9 inhibitor	-
CYP2C9 substrate	–
CYP2D6 inhibitor	—
CYP2D6 substrate	—
CYP3A4 inhibitor	+++
CYP3A4 substrate	+++

Excretion	

CL	3.155
T_1/2_	0.059

Toxicity	

hERG Blockers	—
H-HT	+++
DILI	+++
AMES Toxicity	—
Rat Oral Acute Toxicity	+
FDAMDD	+
Skin Sensitization	—
Carcinogencity	—
Eye Corrosion	—
Eye Irritation	—
Respiratory Toxicity	—
Environmental Toxicity	
Bioconcentration Factors	0.204
IGC_50_	2.854
LC_50_FM	4.322
LC_50_DM	5.041
Tox21 Pathway	
NR-AR	+
NR-AR-LBD	-
NR-AhR	–
NR-Aromatase	—
NR-ER	-
NR-ER-LBD	-
NR-PPAR-gamma	–
SR-ARE	++
SR-ATAD5	—
SR-HSE	—
SR-MMP	-
SR-p53	—
Toxicophore Rules	
Acute Toxicity Rule	0 alert(s)
Genotoxic Carcinogenicity Rule	0 alert(s)
NonGenotoxic Carcinogenicity Rule	1 alert(s)
Skin Sensitization Rule	3 alert(s)

**Table 7:** Observed ADMET features for Metformin.

Medicinal Chemistry	

QED	0.282
SAscore	3.206
Fsp^3^	0.500
MCE-18	0.000
NPscore	-0.278
Lipinski Rule	Accepted
Pfizer Rule	Accepted
GSK Rule	Accepted
Golden Triangle	Rejected
PAINS	0 alert(s)
ALARM NMR Rule	0 alert(s)
BMS Rule	0 alert(s)
Chelator Rule	0 alert(s)

Absorption	

Caco-2 Permeability	-5.745
MDCK Permeability	0.0025
Pgp-inhibitor	—
Pgp-substrate	+++
HIA	—
F_20%_	—
F_30%_	—

Distribution	

PPB	5.598%
VD	1.083
BBB Penetration	–
Fu	76.538%

Metabolism	

CYP1A2 inhibitor	—
CYP1A2 substrate	—
CYP2C19 inhibitor	—
CYP2C19 substrate	—
CYP2C9 inhibitor	—
CYP2C9 substrate	—
CYP2D6 inhibitor	—
CYP2D6 substrate	++
CYP3A4 inhibitor	—
CYP3A4 substrate	—

Excretion	

CL	3.531
T_1/2_	0.369

Physicochemical Property	

Molecular Weight (MW)	129.100
Volume	127.451
Density	1.013
nHA	5
nHD	5
nRot	2
nRing	0
MaxRing	0
nHet	5
fChar	0
nRig	2
Flexibility	1.000
Stereo Centers	0
TPSA	91.490
logS	-1.163
logP	-1.584
logD	-1.471

Medicinal Chemistry	

QED	0.282
SAscore	3.206
Fsp^3^	0.500
MCE-18	0.000
NPscore	-0.278
Lipinski Rule	Accepted
Pfizer Rule	Accepted
GSK Rule	Accepted
Golden Triangle	Rejected
PAINS	0 alert(s)
ALARM NMR Rule	0 alert(s)
BMS Rule	0 alert(s)
Chelator Rule	0 alert(s)

Absorption	

Caco-2 Permeability	-5.745
MDCK Permeability	0.0025
Pgp-inhibitor	—
Pgp-substrate	+++
HIA	—
F_20%_	—
F_30%_	—
Distribution	

PPB	5.598%
VD	1.083
BBB Penetration	–
Fu	76.538%

Metabolism	

CYP1A2 inhibitor	—
CYP1A2 substrate	—
CYP2C19 inhibitor	—
CYP2C19 substrate	—
CYP2C9 inhibitor	—
CYP2C9 substrate	—
CYP2D6 inhibitor	—
CYP2D6 substrate	++
CYP3A4 inhibitor	—
CYP3A4 substrate	—

Excretion	

CL	3.531
T_1/2_	0.369

Toxicity	

hERG Blockers	–
H-HT	++
DILI	–
AMES Toxicity	—
Rat Oral Acute Toxicity	-
FDAMDD	—
Skin Sensitization	++
Carcinogencity	++
Eye Corrosion	—
Eye Irritation	—
Respiratory Toxicity	++
Environmental Toxicity	
Bioconcentration Factors	-0.247
IGC_50_	1.341
LC_50_FM	2.019
LC_50_DM	2.923
Tox21 Pathway	
NR-AR	—
NR-AR-LBD	—
NR-AhR	—
NR-Aromatase	—
NR-ER	–
NR-ER-LBD	—
NR-PPAR-gamma	—
SR-ARE	–
SR-ATAD5	—
SR-HSE	—
SR-MMP	—
SR-p53	—
Toxicophore Rules	
Acute Toxicity Rule	0 alert(s)
Genotoxic Carcinogenicity Rule	0 alert(s)
NonGenotoxic Carcinogenicity Rule	0 alert(s)
Skin Sensitization Rule	0 alert(s)
Aquatic Toxicity Rule	1 alert(s)
NonBiodegradable Rule	1 alert(s)
SureChEMBL Rule	0 alert(s)
FAF-Drugs4 Rule	1 alert(s)

Physiochemical property (Molecular Weight (MW), Volume, Density, nHA, nHD, nRot, nRing, MaxRi ng, nHet, fChar, nRig, Flexibility, Stereo Centers, TPSA, logS, logP, logD); Medicinal Chemistry (QED, SAscore, Fsp^3^, MCE-18, NPscore, Lipinski Rule, Pfizer Rule, GSK Rule, Golden Triangle, PAINS, ALARM NMR Rule, BMS Rule, Chelator Rule); Absorption (Caco-2 Permeability, MDCK Permeability, Pgp-inhibitor, Pgp-substrate, HIA, F_20%,_ F_30%_); Distribution (PPB, VD, BBB penetration, Fu); Metabolism (CYP1A2 inhibitor, CYP1A2 substrate, CYP2C19 inhibitor, CYP2C19 substrate, CYP2C9 inhibitor, CYP2C9 substrate, CYP2D6 inhibitor, CYP2D6 substrate, CYP3A4 inhibitor, CYP3A4 substrate); Excretion (Cl, T_1/2_); Toxicity (hERG Blocker, Respiratory Toxicity, H-HT, DILI, AMES Toxicity, Rat Oral Acute Toxicity,

FDAMDD, Skin Sensitization, Carcinogencity, Eye Corrosion, Eye Irritation); Environmental Toxicity (Bioconcentration Factors, IGC_50_, LC_50_FM, LC_50_DM); Tox21 Pathway (NR-AR, NR-AR-LBD, NR-AhR, NR-Aromatase, NR-ER, NR-ER-LBD, NR-PPAR-gamma, SR-ARE, SR-ATAD5, SR-HSE, SR-MMP, SR-p53); Toxicophore Rules (Acute Toxicity Rule, Genotoxic Carcinogenicity Rule, NonGenotoxic Carcinogenicity Rule, Skin Sensitization Rule).

**Fig. 10:**
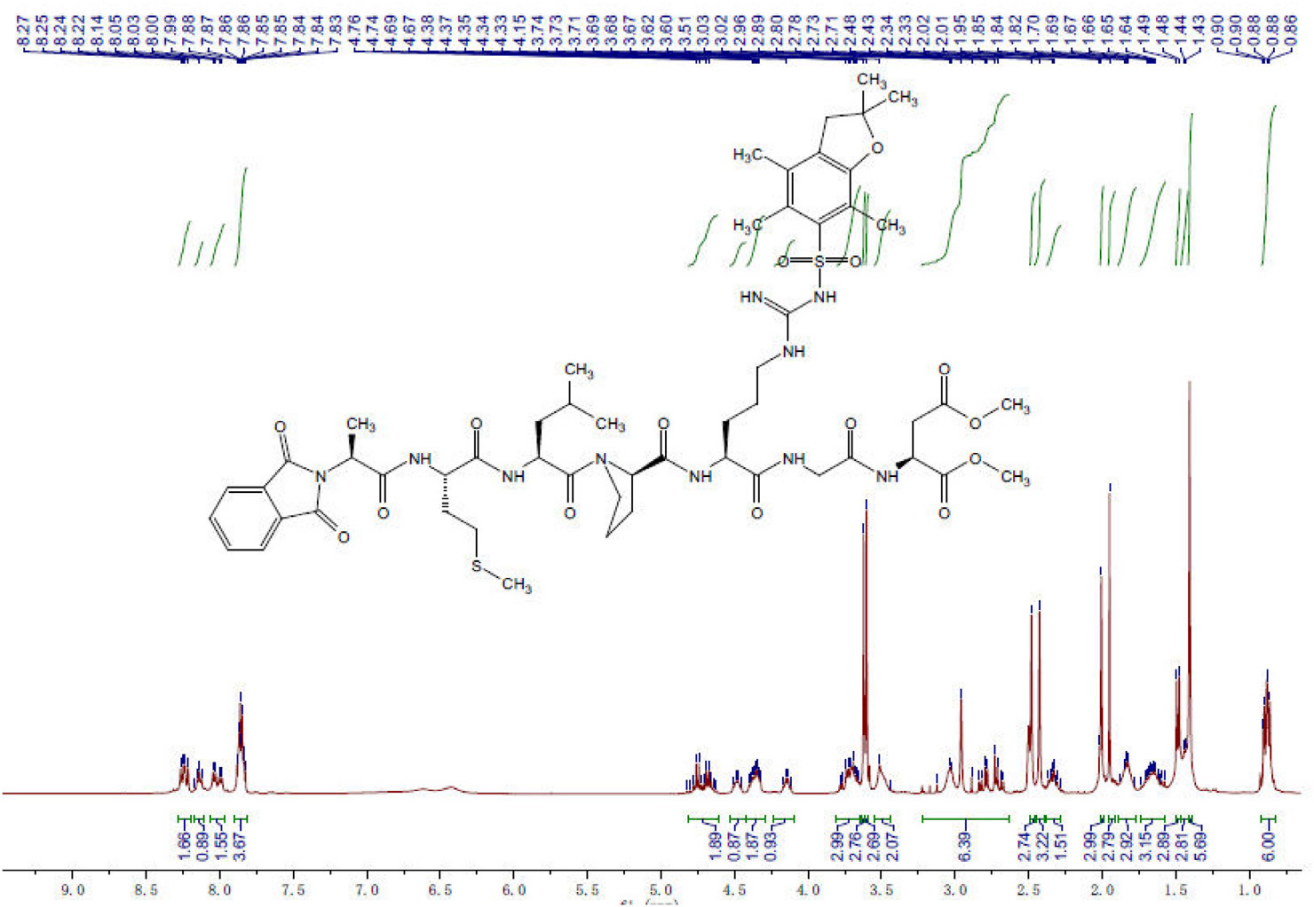
The observed NMR for AB1 (Parent compound).

Fig. 10 showed the signal corresponding to all the protons present in the investigated linear rgd-containing peptides. The chemical shift, coupling constant and number of proton of the investigated compound were ^1^H NMR (400 MHz, DMSO) δ 8.24 (dd, *J* = 12.0, 8.1 Hz, 2H), 8.15 (dd, *J* = 12.0, 6.7 Hz, 1H), 8.02 (dd, *J* = 16.9, 7.2 Hz, 2H), 7.90 – 7.81 (m, 4H), 4.82 – 4.61 (m, 2H), 4.53 – 4.42 (m, 1H), 4.42 – 4.29 (m, 2H), 4.23 – 4.09 (m, 1H), 3.81 – 3.64 (m, 3H), 3.62 (s, 3H), 3.60 (s, 3H), 3.55 – 3.44 (m, 2H), 3.23 – 2.63 (m, 6H), 2.48 (s, 3H), 2.43 (s, 3H), 2.38 – 2.28 (m, 2H), 2.01 (s, 3H), 1.95 (s, 3H), 1.89 – 1.77 (m, 3H), 1.74 – 1.57 (m, 3H), 1.48 (d, *J* = 7.2 Hz, 3H), 1.46 – 1.42 (m, 3H), 1.41 (s, 6H), 0.92 – 0.82 (m, 6H).

## 4 Experimental Design, Materials and Methods

The peptide was synthesized from solid phase peptide synthesis using 2-Chlorotrityl chloride resin. The coupling of the Fmoc-protected amino acids were achieved with Oxyma, NMP and DIC as coupling agent, 20% of piperidine in DMF was used for the deprotection of Fmoc and the peptide was cleaved from resin via 2% trifluoracetic acid in DCM. The crude peptide was esterified with thionyl chloride in methanol to obtain the final product [5], [6], [7].

**Fig. 11:**
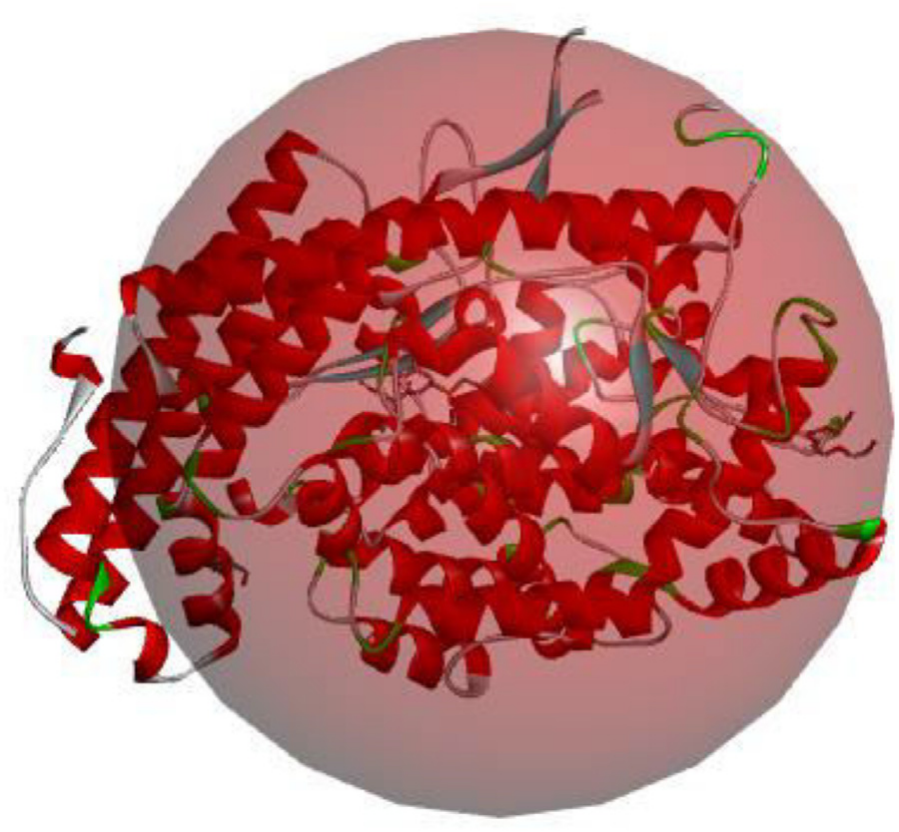
Investigated protein with active site enclosed.

Moreover, the parent compound together with its six derivatives was subjected to optimization using Spartan 14 tool [1,2]. The calculation was set up on core i5, 250GB SSD, 8GB ram and 64-bit operating system, x64-based processor system. The speed for each of the calculation for individual compound was observed to be a function of the size of the compound, the capacity of the system used for the calculation as well as the basis set used. In this work, semi-empirical method was employed and PM3 was used as basis set in the optimization of the investigated compounds. The completion of individual compound brought about series of descriptors with different values except band gap which requires manual calculation (E_LUMO_ – E_HOMO_). The calculated descriptors were located in the properties of the optimized ligand which were retrieved and reported. The ligand (linear rgd-containing peptides) was converted to .pdb format after optimization and further subjected to .pdbqt using AutoDock tool software [8]. The appropriate receptor (angiotensin converting enzyme (PDB ID: 3nxq) [3]) was retrieved from online protein database (protein data bank) and cleaned by removing other materials (water molecules, small ligands etc.) downloaded with the receptor using discovery studio software [9]. The binding site in the downloaded protein (Receptor) was identified using AutoDock Tool [8] and the calculated value obtained for X,Y and Z direction were 3.419222 (center_x), -14.815222 (center_y) and -18.028222 (center_z) while 40, 40 and 40 were used for size_x, size_y and size_z respectively (Fig. 11). The exhaustiveness was set to be 8. The main docking calculation was performed via AutoDock Vina [10]. More so, the correlation between the descriptors and the calculated binding affinities were executed using Microsoft Excel and the correlation with R^2^≥ 0.5 was reported. Compound AB5 (compound with greatest binding affinity) and Metformin were screened for absorption, distribution, metabolism, excretion and toxicity analysis.

## Ethics Statement

This study does not involve studies with animals and humans.

## Declarations

Funding: We are grateful to Bowen University Research Grant Programme for the grant (Grant No: BURG/2023/009).

## CRediT authorship contribution statement

**Abel Kolawole Oyebamiji:** Conceptualization, Methodology, Data curation, Writing – original draft, Visualization, Investigation, Writing – review & editing. **Sunday Adewale Akintelu:** Conceptualization, Methodology, Data curation, Writing – original draft, Visualization, Investigation, Writing – review & editing. **Emmanuel Temitope Akintayo:** Data curation, Visualization, Investigation. **Cecillia Olufunke Akintayo:** Data curation, Writing – review & editing. **Halleluyah O. Aworinde:** Methodology, Data curation, Writing – original draft. **Oluwatobi D. Adekunle:** Methodology, Data curation, Writing – review & editing.

## Data Availability

Dataset on Substituents Effect on Biological Activities of Linear RGD-Containing Peptides as Potential Anti-angiotensin Converting Enzyme (Original data) (Mendeley Data).
